# Atrial Fibrillation in Critically Ill Patients: Incidence and Outcomes

**DOI:** 10.7759/cureus.55150

**Published:** 2024-02-28

**Authors:** Sofia B Paula, André Oliveira, João Melo e Silva, André F Simões, João Gonçalves-Pereira

**Affiliations:** 1 Cardiology Department, Barreiro Montijo Hospital Center, Barreiro, PRT; 2 Intensive Care Department, Hospital Vila Franca de Xira, Vila Franca de Xira, PRT; 3 Faculty of Medicine, Universidade de Lisboa, Lisboa, PRT; 4 Infection and Sepsis Group, Grupo de Investigação e Desenvolvimento em Infeção e Sépsis, Oporto, PRT

**Keywords:** outcome, propensity score match, mortality, critical illness, atrial fibrillation

## Abstract

Background: Atrial fibrillation (AF), either chronic or new onset, is common in critically ill patients. Its epidemiology and relationship with clinical outcomes are poorly known.

Objective: To understand the burden of AF in patients admitted to the ICU and its impact on patients’ outcomes.

Methods: This is a single-center, retrospective cohort study evaluating all patients with AF admitted to a non-cardiac intensive care unit over the course of 54 months. Clinical outcomes were evaluated in the short (hospital discharge) and long term (two-year follow-up). The hazard ratio (HR) with 95% CI was computed for the whole population as well as for propensity score-matched patients, with or without AF.

Results: A total of 1357 patients were screened (59.1% male), with a mean age of 75 ± 15.2 years, length of intensive care unit stay of 4.7 ± 5.1 days, and hospital mortality of 26%. A diagnosis of AF was found in 215 patients (15.8%), 142 of whom had chronic AF. The hospital all-cause mortality was similar in patients with chronic or new-onset AF (31% vs. 28.8%, p = 0.779). Patients with AF had higher in-hospital, one-year, and two-year crude mortality (30.2% vs. 22.9%, p = 0.024; 47.9% vs. 35.3%, p = 0.001; 52.6% vs. 38.4%, p < 0.001). However, after propensity score matching (N = 213), this difference was no longer significant for in-hospital mortality (OR: 1.17; 95% CI: 0.77-1.79), one-year mortality (OR: 1.38; 95% CI: 0.94-2.03), or two-year mortality (OR: 1.30; 95% CI: 0.89-1.90).

Conclusions: In ICU patients, the prevalence of AF, either chronic or new-onset, was 15.8%, and these patients had higher crude mortality. However, after adjustment for age and severity on admission, no significant differences were found in the short- and long-term mortality.

## Introduction

Atrial fibrillation (AF) is characterized by rapid disorganized atrial electrical activation and uncoordinated atrial contraction. It is often associated with structural disease of the heart and its prevalence is increasing [1]. Pre-existing AF is commonly found among older patients with chronic comorbidities, also with a higher prevalence of critical illness and poor prognosis. It is often associated with coronary artery disease, which by itself, is an independent predictor of worse clinical outcomes in ICU patients and seems to contribute to excess mortality [2,3]. New-onset AF can be triggered by accelerated atrial remodeling and arrhythmogenic triggers encountered during critical illness [4] and may be complicated in the long term by chronic AF [5].

It is the most common cardiac arrhythmia in both the general population and the critically ill population [6,7], and has been associated with an increased risk of long-term morbidity, including stroke, dementia, and heart failure, as well as increased mortality [8-10]. However, conflicting results have been reported, probably depending on patients’ comorbidities and different settings [11].

The prevalence of AF in general intensive care units (ICUs) is uncertain. An incidence between 1.8% and 10% in non-cardiac patients admitted to the ICU has been reported [12-14] but more recent data [15,16] found a higher incidence of AF, close to 15%. Moreover, a recent international, multicenter study [15] reported a non-significant higher 90-day mortality. Accordingly, it remains unclear if AF independently impacts prognosis or if it is a marker of underlying severity. Chronic AF is associated with older age, a known risk factor for a worse prognosis [17], and new-onset AF is associated with more severe disease, especially sepsis [15], which also contributes to higher mortality. This is particularly important, as AF treatment itself may contribute to toxicity [18]. We intended to address the prevalence of AF in critically ill patients, both chronic or new-onset in our cohort of ICU patients as well as to evaluate its independent impact on short- and long-term mortality. We also wanted to measure the use of anticoagulation in patients with AF who were discharged alive from the hospital.

## Materials and methods

This was a retrospective, single-center cohort study. We measured the impact of AF, either chronic (paroxysmal, persistent, or permanent) or new onset, on both short-term (hospital mortality) and long-term outcomes (two-year mortality).

We included all consecutive patients admitted to the ICU for over 24 hours between January 2015 and June 2019. Exclusion criteria were age <18 years, or inclusion in another study. Patients were categorized according to the presence or absence of AF, identified through medical records.

Patients’ demographics, comorbidities, type of ICU admission, the presence of sepsis, and the Simplified Acute Physiology Score (SAPS II) [19] on admission were collected along with the ICU length of stay, and the need for organ support therapy. Follow-up was insured for all patients up to two years after hospital discharge, through personal contacts, hospital registries, or the National Health Directory database. We measured the prevalence of AF, both chronic and new-onset. Short and long-term mortality of patients with and without AF were compared.

This study was approved by the Institutional Committee on Human Research of Vila Franca de Xira Hospital at their meeting on 5th January 2023.

Statistical analysis

The features of patients with and without AF were evaluated through descriptive analysis. Continuous variables were reported as mean ± standard deviation (SD) or median (interquartile range) according to data distribution. Categorical variables were reported as percentages.

Baseline demographics and clinical characteristics were compared among patients with AF and without AF, using the Student's T-test or the Mann-Whitney U test for continuous variables, according to data distribution, and chi-square for categorical variables.

A propensity score was calculated for the whole population including 11 variables: age, SAPS II score, comorbidities (heart failure, acute and chronic renal failure, chronic obstructive pulmonary disease, diabetes mellitus, arterial hypertension), gender, sepsis on admission, and type of ICU admission (elective or urgent surgery, medical). A caliper width of 0.05 was used to select a control population, with a 1:1 ratio. Survival up to two years after ICU discharge was assessed through Kaplan-Meier survival curves for the matched population. The log-rank test was computed. The McNemar test was used to assess differences in mortality at hospital discharge, one and two years of follow-up for the whole population, and for the matched cohort. Odds ratios (OR), along with 95% confidence intervals (CIs), were calculated. We also developed a multiple logistic regression analysis, including the whole 1357 patients’ sample, to assess the independent association between AF and two-year mortality. Statistical analysis was performed using IBM SPSS Statistics v.29.0 (IBM Corp., Armonk, NY, USA). All statistics were two-tailed, and the significance level was defined as p < 0.05.

## Results

A total of 1357 patients (59.1% male patients; mean age of 75 ± 15.2 years; ICU length of stay (LOS) of 4.7 ± 5.1 days; hospital mortality of 26%) were admitted to the ICU from January 2015 to June 2019 and were included in the analysis. Their clinical characteristics are presented in Table 1. Of these, 215 patients (15.8%) had a diagnosis of AF, 142 (66.0%) had chronic AF, and 73 had new-onset AF during their ICU stay.

**Table 1:** Baseline clinical characteristics and outcomes of the study population Values are presented as mean ± standard deviation, median (interquartile range), or (%). COPD: chronic obstructive pulmonary disease; ICU: intensive care unit; LOS: length of stay; SAPS II: Simplified Acute Physiology Score II. * Student's t-test. ** Chi-square test. ^#^ Mann-Whitney U test.

	Atrial fibrillation group (N = 215)	Non-atrial fibrillation group (N = 1142)	p-value**
Age (years)	74.5 ± 9.5	64.2 ± 15.5	<0.001*
ICU LOS (days)	5 (3.25-9)	4 (3-8)	0.069^#^
SAPS II	52.7±16.0	44.8±19.3	<0.001*
Male gender (%)	58.6	59.2	0.872
Diabetes mellitus (%)	27	17.8	0.002
Acute renal failure (%)	38.1	30.7	0.038
Chronic renal failure (%)	18.6	7.8	<0.001
Arterial hypertension (%)	44.7	24.6	<0.001
Heart failure (%)	21.9	8.3	<0.001
COPD (%)	2.8	3.5	0.597
Sepsis (%)	47.4	40.6	0.063
Medical admission	76.7	71.5	0.026
In-hospital mortality (%)	30.2	22.9	0.024
1-year mortality (%)	47.9	35.3	0.001
2-year mortality (%)	52.6	38.4	<0.001

No significant differences in short- and long-term mortality were noted between patients with chronic or new-onset AF. However, patients with chronic AF were more often discharged from the hospital receiving anticoagulation (Table 2). Crude short- and long-term mortality was significantly higher in the AF population (Table 1).

**Table 2:** Clinical differences between patients with chronic atrial fibrillation (AF) and new-onset AF Values are presented as mean ± standard deviation, median (interquartile range), or %. AF: atrial fibrillation; ICU: intensive care unit; LOS: length of stay; SAPS II: Simplified Acute Physiology Score II. ^a^ Hospital survivors only. ^*^ Student's t-test. ^**^ Chi-square test. ^#^ Mann-Whitney U test.

	Chronic AF (N = 142)	New-onset AF (N = 73)	p-value**
Age (years)	76.0 ± 8.1	71.6 ± 11.3	0.004*
Male gender (%)	58.5	58.9	>0.99
ICU LOS (days)	6 (4-11)	5 (4-8)	0.04^#^
SAPS II	50.8 ± 27.0	48.5 ± 25.5	0.53*
Sepsis (%)	42.3	57.5	0.043
Arterial hypertension (%)	48.6	37.0	0.113
Heart failure (%)	26.8	12.3	0.015
Anticoagulation at hospital discharge (%)^a^	74.5	25.5	<0.001
In-hospital mortality (%)	31.0	28.8	0.757
1-year mortality (%)	48.6	46.6	0.885
2-year mortality (%)	57.0	43.8	0.083

**Figure 1:**
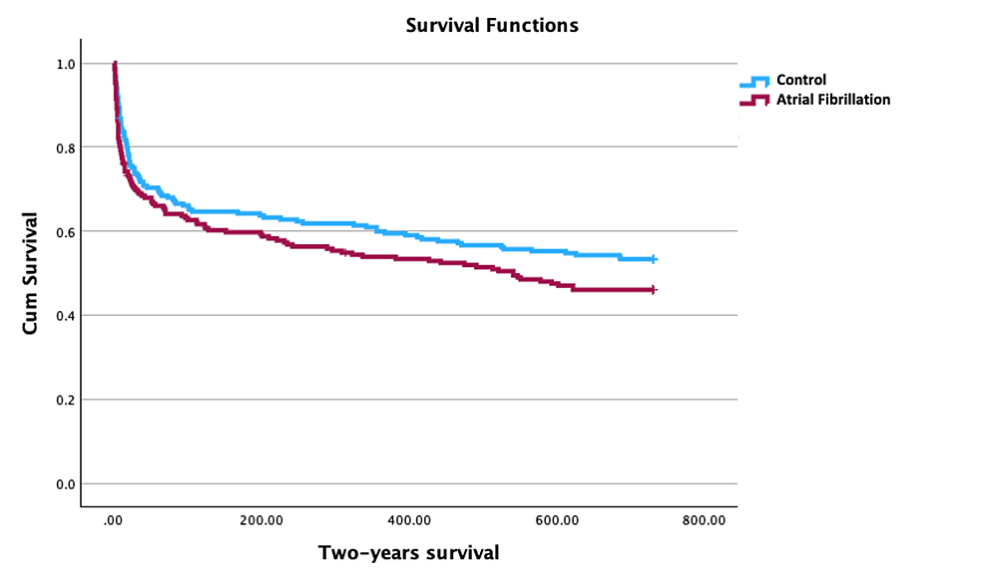
Survival curve up to two years of follow-up Survival curve up to two years of follow-up of patients with atrial fibrillation and the propensity-score matched control group. Log-rank test 2.962 (p = 0.085).

We were able to successfully match 213 patients with AF (99% of our sample) with 213 controls (1:1 ratio). Baseline demographic characteristics were similar between the two matched groups (Table 3). The Kaplan-Meier two-year survival curves are plotted in Figure 1.

**Table 3:** Baseline characteristics and outcomes of patients with atrial fibrillation versus the matched control group Values are presented as mean ± standard deviation or %. COPD: chronic obstructive pulmonary disease; ICU: intensive care unit; LOS: length of stay; SAPS II: Simplified Acute Physiology Score II. * Student's t-test. ** Chi-square test. ^#^ Mann-Whitney U test.

	Atrial fibrillation (N = 213)	Control (N = 213)	p-value**
Age (years)	74.4 ± 9.5	72.1 ± 12.6	0.029^*^
Male gender (%)	58.7	55.4	0.557
Medical admission (%)	76.5	73.2	0.679
Scheduled surgery (%)	6.6	8.5	
Unscheduled surgery (%)	16.9	18.3	
SAPS II	52.7 ± 16.1	50.7 ± 18.1	0.225^*^
Diabetes mellitus (%)	27.2	24.4	0.580
Acute renal failure (%)	38.5	41.3	0.621
Chronic renal failure (%)	18.3	19.2	0.901
Arterial hypertension (%)	44.1	39.9	0.432
Heart failure (%)	21.1	24.4	0.488
COPD (%)	2.8	5.6	0.228
Sepsis (%)	47.9	37.6	0.040
ICU LOS (days)	5 (4-9)	5 (3-8)	0.700^#^
Hospital mortality (%)	30.5	27.2	0.521
1-year mortality (%)	48.4	40.4	0.119
2-year mortality (%)	53.1	46.5	0.208

In the propensity score-matched group, no significant difference in mortality was noted, neither at hospital discharge (30.0% vs. 28.2%; OR: 1.10; 95% CI: 0.72-1.66; p = 0.454), at one year of follow-up (47.9% vs. 44.1%; OR: 1.16; 95% CI: 0.79-1.70; p = 0.1), or at two years of follow up (53.1% vs. 49.3%; OR: 1.16; 95% CI: 0.79-1.70; p = 0.45).

**Figure 2:**
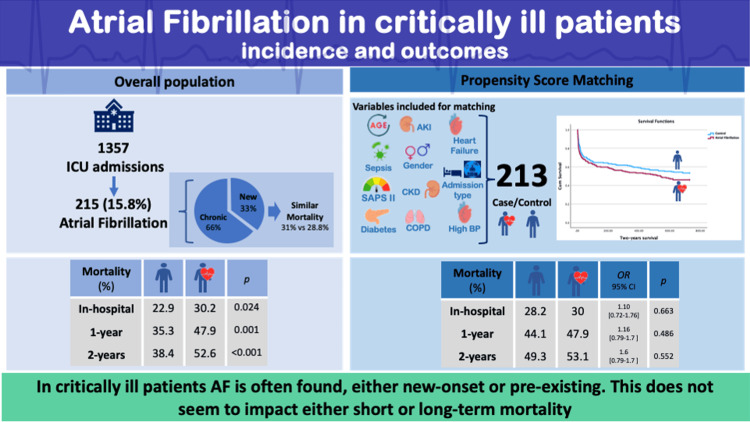
Graphical abstract AF: atrial fibrillation; AKI: acute kidney injury; SAPS II: Simplified Acute Physiology Score II; CKD: chronic kidney disease; COPD: chronic obstructive pulmonary disease; BP: blood pressure. Image created by the authors.

Similar results were found in the multiple logistic regression analysis, with no independent association between AF and two-year mortality (OR: 0.84; 95% CI: 0.60-1.18; p = 0.318) (Table 4 and Figure 2).

**Table 4:** Multivariate regression model forecasting two-year mortality Variables included in multivariate logistic regression forecasting two-year mortality model. COPD: chronic obstructive pulmonary disease; ICU: intensive care unit; LOS: length of stay; SAPS II: Simplified Acute Physiology Score II.

Variable	Odds ratio	95% CI	p-value
Age (years)	1.02	1.01-1.03	<0.001
Male gender	1.03	0.80-1.31	0.83
Admission type	0.77	0.58-1.03	0.077
SAPS II	1.04	1.03-1.05	<0.001
Atrial fibrillation	0.84	0.60-1.18	0.318
Diabetes mellitus	0.92	0.67-1.25	0.58
Acute renal failure	1.25	0.95-1.63	0.107
Chronic renal failure	0.96	0.62-1.43	0.79
Arterial hypertension	1.36	1.02-1.80	0.034
Heart failure	1.05	0.71-1.56	0.814
COPD	1.44	0.73-2.84	0.29
Sepsis	0.91	0.71-1.16	0.443

## Discussion

In this study, we presented the results of a single-center, retrospective, cohort study of patients admitted to a non-cardiac, medical-surgical ICU. We assessed short and long-term (two years) follow-up of the whole population.

We found a high prevalence of AF (15.8%) aligned with previously published data [20,21]. Of the patients with AF, two-thirds already had this diagnosis before being admitted to the ICU, while one-third had new-onset AF. Although these populations may differ, in our study, hospital and two-year mortality rates of both populations were similar.

A recently published prospective multicenter study noted a high prevalence of new-onset AF (13.3%) [15], with almost the same number of persistent or permanent AF. Patients with new-onset AF had higher 90-day mortality (41.2 vs. 25.2%), although not significant after adjustment [15], which may be related to the high burden of cardiovascular comorbidities, frequently present in this group [18]. Chronic AF is more often associated with older age, a well-known risk factor for mortality [17]. On the other hand, new-onset AF is more related to acute insult, especially sepsis [15]. In our population, we also found differences in mortality and the distribution of these risk factors, which may have balanced one another and contributed to a similar SAPS II score (Table 2) and outcome.

In our study, patients with AF had higher mortality throughout their follow-up (Table 1) and roughly 50% died after two years. To evaluate the independent impact of AF on these outcomes, we matched AF patients with a similar control group, to account for potential confounders, using a propensity score [22]. After adjusting for confounding factors, patients with AF (either chronic or new-onset AF) had similar short and long-term outcomes at hospital discharge and after one and two years of follow-up (Figure 1).

In a large multicenter cohort study (FROG-ICU) [23], AF was associated with increased 90-day all-cause mortality, but only for patients with new-onset AF (23.6% vs. 15.3%; adjusted HR: 1.37, 95% CI: 1-26-1.5, p < 0.001). However, these differences were mostly related to patients who were admitted to a coronary ICU, a population that was not included in our study, and their illness severity, presence of sepsis, and mean ICU length of stay were much lower than in our cohort. Interestingly, a single-center retrospective study by Moss et al. [24] showed that only patients with chronic AF (and not new-onset AF) had lower survival rates.

In our cohort, less than half of the patients received anticoagulation at hospital discharge (39.5%) and, among them, patients with previous AF were more likely to be anticoagulated. Our study design does not allow us to address the individual patient’s embolic or hemorrhagic risks. Of note, Jacobs et al. did not find a benefit in the one-year survival of the 56.3% of patients with new-onset AF, who received anticoagulation at hospital discharge [25]. The role of anticoagulation in these patients, especially new-onset AF during the ICU stay, is not well established [18-20]. A possible explanation could be the link between new-onset AF and illness severity, although an AF recurrence rate of 50%, associated with myocardial remodeling, has also been proposed [4].

Limitations

This was a single-center retrospective study. The diagnosis of AF was based on medical records and recognition of this diagnosis during the ICU stay was not ensured. Moreover, the therapeutic approach of patients with AF was not protocolized and unidentified bias may have occurred. We did not collect data on some relevant comorbidities, such as structural heart disease. Finally, we may have missed patients with AF episodes that were not registered (mainly short-term, spontaneously resolving episodes), and it was not possible to measure the duration and frequency of tachyarrhythmic events.

## Conclusions

In our cohort of 1357 critically ill patients, AF was present in 15.8%. Patients with AF, either chronic (2/3) or new-onset, had similarly high short- and long-term mortality, reaching more than 50% after two years of follow-up. Crude mortality was much higher in patients with AF, but this seemed to be related to the burden of comorbidities. After adjustment for age and severity on admission, there were no significant differences in mortality between patients with or without AF.

Also, despite a very different rate of anticoagulation at discharge between chronic and new-onset AF patients, we did not find any substantial impact of this therapy on the long-term outcome.
